# Exosomes and Atherogenesis

**DOI:** 10.3389/fcvm.2021.738031

**Published:** 2021-08-26

**Authors:** Bingbing Lin, Juan Yang, Yuwei Song, Guohui Dang, Juan Feng

**Affiliations:** ^1^Key Laboratory of Molecular Cardiovascular Science, Ministry of Education, Department of Physiology and Pathophysiology, School of Basic Medical Sciences, Peking University, Beijing, China; ^2^Department of Integration of Chinese and Western Medicine, School of Basic Medical Sciences, Peking University, Beijing, China

**Keywords:** exosome, vascular injury, inflammation, immunocyte, atherosclerosis

## Abstract

Myocardial infarction and ischemic stroke are the leading causes of mortality worldwide. Atherosclerosis is their common pathological foundation. It is known that atherosclerosis is characterized by endothelial activation/injury, accumulation of inflammatory immune cells and lipid-rich foam cells, followed by the development of atherosclerotic plaque. Either from arterial vessel wall or blood circulation, endothelial cells, smooth muscle cells, macrophages, T-lymphocytes, B-lymphocytes, foam cells, and platelets have been considered to contribute to the pathogenesis of atherosclerosis. Exosomes, as natural nano-carriers and intercellular messengers, play a significant role in modulation of cell-to-cell communication. Under physiological or pathological conditions, exosomes can deliver their cargos including donor cell-specific proteins, lipids, and nucleic acids to target cells, which in turn affect the function of the target cells. In this review, we will describe the pathophysiological significance of various exosomes derived from different cell types associated with atherosclerosis, and the potential applications of exosome in clinical diagnosis and treatment.

## Introduction

According to epidemiological investigation, the number of deaths from atherosclerotic cardiovascular disease (ASCVD) in 2016 was about 2.4 million, accounting for 61% of cardiovascular deaths and 25% of total deaths (1). Moreover, ASCVD mortality has increased significantly over the past 30 years. Atherosclerosis, a progressive multifactorial degenerative disease of large and medium arterial walls (2–4), is the root cause of most cardiovascular diseases including coronary artery disease (CAD), ischemic gangrene, abdominal aortic aneurysm, heart failure and ischemic stroke (5). It is known that atherosclerosis is a complex multifactorial disease developed through a series of events involving the cardiovascular system, metabolism, and immune system (6, 7). Increasing evidence suggests that local inflammatory microenvironment consisting of different inflammatory cells is a fundamental pathological characteristic (2, 8–11), in which cells exchange information through different mechanisms and structures, such as secreting bioactive molecules (growth factors, chemokines, peptides, ions, bioactive lipids, and nucleotides), direct cell-cell contact and cell-matrix interaction (12, 13). In the past few years, extracellular vesicles (EVs) derived from various cells have received increasing attention (14). EVs are membranous vesicles abounding in body fluids. There are three types of EVs, including exosomes, microvesicles (MVs) and apoptotic bodies. Among them, exosomes have been paid increasing attention and been considered as a mediator for cellular communication, which can deliver a series of bioactive components to target cells and then modulate their functions (12, 15). This review will focus on the sources of different exosomes and elucidate how they contribute to the pathogenesis of atherosclerosis, eventually provide evidence to suggest their potential clinical applications.

## Introduction of Exosomes

Exosomes used to be considered as “extracellular debris” and received little attention. Now exosomes are generally recognized as a mediator of cell-to-cell communication which are the smallest kind of extracellular vesicles (30–150 nm) with bilayer membrane morphology, and usually in the shape of cup (16).

Exosomes were first described as microvesicles containing 5′-nucleotidase activity released by tumor cell lines (17). In 1983 and 1985, Harding et al. (18) and Pan et al. (19) respectively reported and proved that cultured reticulocytes released exosomes. Using immunoelectron microscope, they saw the dynamic change process of internalized anti-transferrin receptor antibody in the reticulocytes. The released vesicles were separated and purified, and then named after “exosomes”. Afterwards, it has been confirmed that apart from reticulocytes, EVs can be secreted from various types of living cells, such as B lymphocytes, macrophages and endothelial cells (14). Moreover, exosomes can be extracted from different body fluids, including blood, ascites, cerebrospinal fluid, saliva, breast milk and urine (16).

To this day, although there is still no standardized method for exosome isolation, many techniques have been established based on the biochemical and physicochemical features of exosomes (20). The exosome separation methods include: ultracentrifugation, ultrafiltration, immunoaffinity capture, charge neutralization-based polymer precipitation, size-exclusion chromatograph, and microfluidic techniques (20). Each one has its unique advantages and disadvantages. Now one of the most common used strategies is ultracentrifugation (21). In recent years, microfluidic techniques are attracting more and more attention for their simplicity, fast-isolating and material-saving features, which may be commonly applied to exosome separation and detection in the future (20).

Exosomes can be formed in two mechanisms: the ESCRT-dependent and ESCRT-independent mechanisms, contribution of which may vary depending on the contents recruited and the type of donor cells (22). According to the current database of the exosome contents, 4,563 proteins, 1,639 mRNAs, 764 miRNAs, and 194 lipids have been identified in exosomes (23). The nucleic acids, proteins and other molecules carried by exosomes give exosomes rich biological information, which is transmitted from donor cells to target cells to play a specific biological effect (24). In addition, exosomes are enriched in membrane markers, such as tetraspanins and endosomal sorting complex required for transport members (25, 26). Furthermore, exosomes can also serve as biomarkers and potential targets for diagnosis and treatment of ASCVD (27, 28).

## The Roles of Exosomes in the Occurrence and Development of Atherosclerosis

Atherosclerosis is a multifactorial disease closely associated with endothelial cell damage, vascular inflammation, accumulation of monocytes and macrophages, and thrombosis (29). Exosomes from endothelial cells, smooth muscle cells, macrophages, T-lymphocytes have been considered to contribute to the atherosclerosis-related pathological processes, such as inflammation, vascular injury, calcification, apoptosis, thrombosis, and coagulation (22, 30).

### The Roles of Monocytes/Macrophages Exosomes in Atherosclerosis

In the early stages of atherosclerosis, inflammation induces the expression of vascular endothelial cell adhesion molecules and vascular cell chemokines, recruiting circulating monocytes. These monocytes move into the lumen of arteries and then differentiate into macrophages in response to local mediators such as mononuclear colony stimulating factors (10). Monocyte-derived macrophages are recruited, differentiate and proliferate continuously and thus become the main group of cells involved in the formation of atherosclerotic plaques. Monocytes and macrophages can secret abundant exosomes, mainly found in atherosclerotic lesion (6, 31). Exosomes derived from monocytes are key players in inflammation. By interacting with endothelial cells, these exosomes induce expression and activation of adhesion molecules and pro-inflammatory cytokines. Meanwhile, they can also interact with other types of blood vessel cells, including monocytes, fibroblasts and smooth muscle cells and then regulate immune response and vascular inflammation microenvironment (32, 33).

#### Monocyte Exosomes Promote Vascular Inflammation, Endothelial Cell Apoptosis and Thrombosis

Friedrich et al. isolated exosomes from starved human monocyte cell line (THP-1) and then injected intravenously into ApoE-/- mice fed on a high-fat and high-cholesterol diet. The results showed that monocyte exosomes significantly increased monocyte and T lymphocyte infiltration in mouse vascular wall and enhanced plaque formation. The results of *in vitro* cell culture experiments confirmed that the uptake of monocyte exosomes triggered the production of reactive oxygen species and the release of proinflammatory interleukin-6 in macrophages, as well as macrophage migration regulated by monocyte chemotactic protein 1 (MCP-1). After co-incubation with endothelial cells, monocyte exosomes can also significantly increase the expression of intracellular adhesion molecule-1 (ICAM-1) in monocytes. Therefore, monocyte exosomes may exacerbate vascular inflammation via paracrine during atherosclerosis (34).

During the pathogenesis of atherosclerosis, oxidized lipoprotein (ox-LDL) promotes the adhesion of monocytes to intima by inducing the expression of adhesion molecules of endothelium, thereby promoting the formation and progression of lesions. Chen et al. reported that exosomes secreted by ox-LDL-treated THP-1 cells, were rich in long non-encoding lncRNA GAS5. These exosomes could promote apoptosis of vascular endothelial cells when they transferred to the perinuclear region. In contrast, exosomes released by THP-1 cells knockout of lncRNA GAS5 inhibited endothelial cell apoptosis and expression of P53, Caspase 3, Caspase 7, and Caspase 9 (35). To understand the direct effect of monocyte exosomes on endothelial cells in the NO pathway, Maria et al. treated THP-1 cells with the apoptotic agent epotoside (VP-16). They discovered that the exosomes secreted by these cells could promote the production of NO in human endothelial cells by inhibiting the expression of caveolin-1 in the pits of endothelial cells. The inhibition of caveolin-1 expression by exosomes could be reversed by inhibiting phosphatidylinositol-3-kinase (PI-3-K) or mitogen-induced extracellular kinase 1/2 (MEK1/2). These results suggest the pleiotropic effect of monocyte microparticles on the function of vascular endothelial cells and dissect the related signaling pathways (36).

Thrombosis of atherosclerotic plaque depends largely on the morphology of the plaque and relative levels of tissue factor (TF) and TF pathway inhibitor (TFPI) (37). Monocyte-derived exosomes are considered potent coagulants for they contain TF that leads to the formation of procoagulant thrombin (38). Manjunath et al. found that exosomes from circulating monocytes carried more TF in lipid-rich carotid atherosclerosis. These exosomes improved the risk of thrombosis by inducing imbalances in TF and TFPI levels in vessels (39).

#### The Roles of Macrophage Exosomes in Endothelial Cell Migration, Monocyte Infiltration, Foam Cell Aggregation, and Vessel Calcification

During inflammation, endothelial cell migration and leukocyte recruitment are strictly controlled by integrin activation and internalization, which is a critical step in the process of angiogenesis and is closely related to the formation of atherosclerosis (40, 41). In 2014, Lee et al. found that exosomes derived from human macrophages could inhibit the migration of endothelial cells by regulating intracellular integrin β1 trafficking. First, the exosomes secreted from macrophages promoted the internalization of integrin β1 in primary human umbilical vein endothelial cells (HUVECs). Integrins accumulated in the perinuclear region rather than recycled back to the plasma membrane, leading to the proteolytic degradation of integrin. Second, macrophage-derived exosomes enhanced ubiquitination degradation of HUVECs integrin β1. Third, macrophage-derived exosomes inhibited the migration of HUVECs by inhibiting integrin β1-regulated collagen-induced mitogen-activated protein kinase/extracellular kinase signaling pathway (42). In addition, ox-LDL-stimulated macrophages might attenuate the growth and tube formation of endothelial cells and exosomes derived from these macrophages might be involved in the process (43).

Monocyte infiltration is closely related to the formation of early lesions in atherosclerosis. Damaged endothelial cells release growth factors to recruit monocytes. Monocytes adhere to the endothelium, and then migrate into the sub-endothelial space, continuously taking up ox-LDL in the intima and convert to foam cells. Tang et al. demonstrated that exosomes released from LPS-treated macrophages increased the expression of adhesion molecules ICAM-1, chemokine ligand CCL-2 and cytokine IL-6 in human vascular endothelial cells, which induced monocyte adhesion and migration (44). In 2017, Osada-Oka et al. found that in addition to ICAM-1, macrophage-derived exosomes could also promote the expression of plasminogen activator inhibitor-1 (PAI-1) and increase monocyte infiltration (45).

Foam cells derived from monocytes and smooth muscle cells accumulate and form fatty streak lesions. Fat streaks are the earliest lesions visible to the naked eye in atherosclerosis and exist through the development of plaques. Studies have shown that the release of galectin-3 (Gal-3) in macrophage- and monocyte- derived exosomes is regulated by the intercellular redox reaction. Gal3 may increase intracellular cholesterol accumulation by regulating macrophage endocytosis of low-density lipoprotein, increasing the formation of foam cells (46). In addition, miR-146a in exosomes derived from cholesterol-loaded macrophages could inhibit the expression of the pro-migratory genes IGF2BP1 and HuR in macrophages *in vitro* and *in vivo* (47). Obstruction of macrophage migration led to further accumulation of foam cells, resulting in the spread of inflammation and instability of atherosclerotic plaques. The fatty streaks are followed by fibrous lesions, which are characterized by the formation of necrotic debris and the presence of VSMCs. Niu et al. established macrophage-derived foam cell models and found that these foam cells released more vesicles than normal macrophages. Proteomic data analysis indicated that foam cell-derived exosomes promoted adhesion and migration of VSMCs by regulating the actin and focal adhesion pathways of the cytoskeleton. Western blot results showed that foam cell-derived vesicles could transport proteins to VSMCs, thereby activating ERK and Akt pathways in VSMCs and promoting adhesion and migration of VSMCs, which might accelerate the development of atherosclerotic plaques (48).

Microcalcification of the thin coronary arteries covering the intimal necrosis of atherosclerotic plaque can lead to plaque rupture, causing acute cardiovascular events (49). Vascular calcification is a ubiquitous phenomenon in atherosclerotic plaque and an inevitable result of atherosclerosis. There are abundant macrophages in the early microcalcification plaques (50). Sophie et al. found that proinflammatory macrophages release pro-calcified exosomes by the phosphatidylserine-membrane adhesion protein 5-S100A9 membrane complex. These exosomes rich in membrane adhesion proteins 5 and S100A9 directly promotes microcalcification (51).

In addition, the study of Yong-Gan Zhang et al. indicated that exosomal miR-146a derived from oxLDL-treated macrophage could promote the overproduction of ROS and NETosis by decreasing SOD2 expression in neutrophils, leading to atherosclerosis deterioration (52). A recent study discovered that Nicotine might induce atherosclerotic lesion progression after administered to ApoE^−/−^ mice. Macrophage-derived exosomes containing miR-21-3p might play a role in the process of plaque progress by increasing VSMC migration and proliferation through its target PTEN (53).

In conclusion, monocyte/macrophage-derived exosomes promote atherosclerosis mainly by inhibition of vascular endothelial cell migration and promotion of endothelial cell apoptosis, monocyte infiltration, inflammation, oxidation and vascular microcalcification.

### The Roles of Endothelial Cell Exosomes in Atherosclerosis

Recent studies have shown that endothelial cell-derived exosomes mediated the interactions among endothelial cells, smooth muscle cells, and macrophages and played an important role in the pathogenesis of atherosclerosis (54). A total of 1,354 proteins and 1,992 mRNA were found in the vesicles released from cultured normal endothelial cells, reflecting the complexity of vesicle transport in endothelial cells (55).

#### The Roles of Endothelial Cell Exosome in Vessel Homeostasis

The cargo delivered by exosomes can regulate cell survival/death, inflammation and tumor metastasis. Therefore, exosomes are capable of modulating angiogenesis and thus play a role in maintaining vessel homeostasis.

Kruppel-like factor 2 (KLF2) is a shear stress-induced transcription factor with protective effects against atherosclerosis (56). HUVECs could secrete extracellular vesicles rich in miR-143/145 through KLF2-mediated or shear stress-stimulated mechanism. These vesicles could regulate the phenotype of smooth muscle cells and inhibit the dedifferentiation of human aortic smooth muscle cells, which is one of the reasons for its atheroprotective effect in mice (57). Bas et al. showed that miR-214 could control endothelial cell function and angiogenesis, and play a leading role in exosome-mediated signaling between endothelial cells. Endothelial cells released exosomes containing miR-214, which could stimulate angiogenesis by silencing the expression of ataxia telangiectasia mutated in adjacent target cells (58). In addition, IL-3 increased vascular endothelial cell-activated signal transduction and transcriptional activator 5 (pSTAT5)-mediated release of exosomes containing miR-126-3p and pre-miR-126, thereby promoting angiogenesis (59).

Angiogenesis and homeostasis are complex biological processes that are strictly controlled by multiple signaling pathways. Among them, the angiopoietin-Tie2 signaling pathway has received increasing attention in the past decade (60). Angiopoietin-2 (Ang2), involved in the regulation of vascular homeostasis and vascular integrity, is an extracellular protein produced primarily by endothelial cells and the main ligand of the Tie2 receptor (61). Ju et al. demonstrated that Ang2 was secreted by endothelial cells through exosomes, which was regulated positively by the syndecan 4/syntenin signaling pathway and negatively by PI3K/Akt/endothelium-dependent nitric oxide synthase (eNOS) signaling. The vascular defects observed in Akt-/- mice were partly due to the excessive secretion of Ang2 and could be ameliorated by syndecan-4 knockdown that reduced the level of extracellular Ang2. This suggested that three key signaling pathways, including angiopoietin/Tie2, PI3K/Akt/eNOS and syndecan/syntenin, played an important role in blood vessel growth and stabilization (62). Delta-like 4 (Dll4) is a Notch ligand which is expressed in endothelial cells and up-regulated during angiogenesis. As reported, endothelial exosomes incorporated with Dll4 protein could transfer Dll4 to other endothelial cells, resulting in low Notch signaling and injure the vascular integrity (63).

#### The Roles of Endothelial Cell Exosomes in Vascular Inflammation

High concentrations of ox-LDL and homocysteine (Hcy) are independent risk factors of atherosclerosis and coronary heart disease (64, 65). High level of circulating heat shock protein 70 (HSP70) is also at risk of vascular disease (66). Zhan et al. found that Ox-LDL and Hcy enhanced the release of HSP70-containing exosomes from rat arterial endothelial cells. These extracellular HSP70 cannot directly activate endothelial cells, but can recruit circulating monocytes to adhere to vascular endothelial cells, and thus participating in the pathogenesis of vascular inflammation (54, 67).

Long non-coding RNA-RNCR3 was significantly upregulated in mouse and human aortic atherosclerotic lesions and RNCR3 knockdown could accelerate the development of atherosclerosis and releases of inflammatory factor. Endothelial cell derived-exosomal RNCR3 could exert a remarkable atheroprotective effect *via* mediating the communication between ECs and VSMCs (68). Ten-eleven translocation 2 (TET2), a member of methylcytosine dioxygenase is considered as a key molecular to switch the phenotype of VSMCs. In response to pro-inflammatory stimuli, the CD137 pathway was activated in endothelial cells and the TET2 was reduced in endothelial cell-derived exosomes, which transferred into and then induced VSMC phenotype switch, promoting plaque formation and AS development (69).

Vesicles from TNF-α-induced inflammatory vascular endothelial cells were easily taken up by THP-1 and HUVECs. Compared with the control, the vesicles derived from inflammatory vascular endothelial cells contained intercellular adhesion molecules and chemokines, including ICAM-1, CCL-2, IL-6, IL-8, CXCL-10, CCL-5 and TNF-α. The vesicles could mediate the selective transfer of functional inflammatory mediators to target cells and modulate them to pro- or anti-inflammatory mode. Inflammatory vascular endothelial cell-derived vesicles could increase the expression of pro-inflammatory cytokines IL-6, IL-8 and ICAM-1 in HUVECs. These vesicles also increased the expression of pro-inflammatory ICAM-1 and CCL-4 in THP-1, as well as the expression of pro- and anti-inflammatory CCL-5 and CXCL-10 proteins, thereby inducing the macrophages to exhibit both pro-inflammatory and anti-inflammatory phenotypes. At the functional level, endothelial cell exosomes mediated inflammation and promoted adhesion and migration of THP-1. These evidences suggest that exosomes released by inflammatory endothelial cells are rich in a variety of inflammatory markers, chemokines, and cytokines that establish targeted cross-talk between endothelial cells and monocytes and reprogram them to pro- or anti-inflammatory phenotype (70).

HUVECs released a large quantity of exosomes rich in inflammation-associated miR-155 after ox-LDL stimulation. Later, miR-155 was transferred to THP1 cells by exosomes to promote the polarization of the anti-inflammatory M2 macrophages to pro-inflammatory M1 macrophages. The vesicles expressing KLF2 secreted from endothelial cells inhibited monocyte activation and decreased inflammation. Oil red O staining showed that exosomes secreted by endothelial cell expressing KLF2 significantly reduced atherosclerotic lesions in mice, decreased pro-inflammatory M1 macrophages and increased anti-inflammatory M2 macrophages, which partly resulted from the down-regulation of miR-155 expression (71).

Neutrophil extracellular traps (NETs) play an important role in the pathological process of atherosclerosis. Both exosomes from atherosclerosis patients and ox-LDL treated HUVECs induced NETs release from neutrophils, aggravating atherosclerosis. The highly-expressed miR-505 encapsulated in these exosomes might inhibit Sirtuin 3 (SIRT3) in neutrophils, increasing ROS levels and NET release by neutrophils (72). Metastasis-associated lung adenocarcinoma transcript 1 (MALAT1), a long non-coding RNA has been widely shown to be involved in tumorigenesis, but the role of exosomal MALAT1 in atherosclerosis is likely to be controversial. Gao H et al. came to the conclusion that exosomal MALAT1 derived from ox-LDL-treated HUVECs promoted the formation and release of NETs which might further deteriorated atherosclerosis (73). However, Hongqi L et al. found that the MALAT1 expression in the exosomes derived from ox-LDL treated HUVECs was lower than the exosomes derived from untreated HUVECs. Their further *in vivo* experiments showed the loss of MALAT1 from ox-LDL-VECs-Exos in mouse repressed the nuclear factor erythroid 2-related factor (NRF2) signaling pathway and failed to inhibit dendritic cells maturation, which might be associated with atherosclerosis progression (74). Therefore, the precise roles of exosomal MALAT1 in atherogenesis should be further investigated.

### The Roles of Platelet-Derived Exosomes in Atherosclerosis

Platelets are involved in thrombosis. Platelet activation and endothelial damage play an important role in atherosclerosis. A variety of platelet-derived exosomes regulate the progression of atherosclerosis (75, 76).

Various agonist-activated platelets release exosomes from plasma membrane. Orla P. Barry et al. found that activated platelets increased monocyte adhesion to HUVECs in a time- and dose-dependent manner, primarily due to the exosomes secreted by these platelets selectively increasing the expression of the adhesion molecule ICAM-1 and inflammatory cytokines including IL-1β, IL-6, and IL-8 in HUVECs while having no up-regulating effects on the expression of adhesion molecules VCAM-1 and P-/E-selectin (77). Furthermore, in 2003, Kaneider et al. found that thrombin-activated platelet exosomes carried CD40 ligands and triggered dendritic cell maturation through the mechanism of CD40 ligands to modulate inflammatory immune responses (78).

Exosomes secreted by thrombin-activated platelets have protective effects on atherosclerotic endothelial inflammation. The miR-223 was elevated in thrombin-activated platelet exosomes and was transferred into vascular endothelial cells to inhibit phosphorylation of p38, JNK and ERK, then blocked nuclear translocation of p65 of NF-κB, ultimately decreased the high expression of ICAM-1 stimulated by TNF-α (79). MiR-223 in platelet exosomes also regulated apoptosis of vascular endothelial cells and affected the development of atherosclerosis. miR-223 was significantly increased in platelet-derived exosomes stimulated by thrombopoietin or thrombin and in patients with atherosclerosis. Then exosomes introduced miR-223 into HUVECs, which in turn reduced insulin-like growth factor 1 receptors and ultimately promoted apoptosis of HUVECs induced by advanced glycation end products (80). In addition, Yao Y et al. discovered that exosomes derived from platelets of the atherosclerosis models of ApoE-/- mice exhibited high expression level of miR-25-3p which could target Adam 10 and reduce its expression in the ox-LDL-treated coronary vascular endothelial cells (CVECs), leading to the attenuation of CVEC inflammation (81).

Platelet-derived exosomes can also inhibit platelet aggregation *in vitro* and reduce the adhesion of platelets to exposed collagen from damaged blood vessels caused by high shear forces *in vivo*. By decreasing the reactivity of platelets, exosomes inhibit vascular occlusive thrombosis in a model of damaged arterial caused by ferric chloride. In atherosclerosis, exosomes from activated platelets significantly reduce type II scavenger receptor CD36 expression in platelets, thus reducing platelet aggregation. On the other hand, exosomes from activated platelets also inhibit uptake of ox-LDL in macrophages, thereby preventing the formation of foam cells by reducing the expression of type II scavenger receptor CD36 in macrophages, ultimately inhibiting thrombosis in atherosclerosis (82).

In summary, activated platelet-derived exosomes play an important regulatory role in endothelial damage, endothelial cell apoptosis, monocyte adhesion, and dendritic cell maturation during atherosclerosis. At the same time, platelet-derived exosomes inhibit the development of atherosclerosis by inhibiting platelet aggregation and thrombosis. However, the *in vivo* regulatory mechanism still needs further studies.

### The Roles of Vascular Smooth Muscle Cell Exosomes in Atherosclerosis

Smooth muscle cells (SMCs) differentiation and endothelial cell damage promote the formation of atherosclerotic plaques (83). Exosomes derived from VSMCs mediate KLF5-induced miR-155 transfer from SMCs to endothelial cells, thereby destroying endothelial tight junctions and barrier integrity, increasing endothelial permeability and promoting atherosclerosis (84). The transfer of miR-155 to endothelial cells leads to the overexpression of miR-155 in endothelial cells, which can inhibit proliferation, migration and re-endothelialization of endothelial cells *in vitro* and *in vivo*, thereby increasing permeability of vascular endothelium (85).

Alexander et al. used calcified cultured human VSMCs as a model of atherosclerotic vascular calcification. Exosomes released by these VSMCs contained high calcium and extracellular matrix proteins. Extracellular high calcium induces expression of sphingomyelin diesterase 3 and release of VSMC-secreted high calcium-contained exosomes *in vitro*. After VSMCs were stimulated by tumor necrosis factor-α and platelet-derived growth factor-BB, exosomes were released and deposited in blood vessels, thereby accelerating the calcification of blood vascular walls (86).

In the diabetic mouse model, exosomes derived from VSMCs containing miR-221/222 were more likely to cause atherosclerosis compared with the non-diabetic mice, and the introduction of exosomes of diabetic VSMCs into ApoE-/- mice led to deterioration of atherosclerotic lesions (87).

Thrombosis caused by atherosclerotic plaque rupture is the main cause of vascular embolism events including myocardial infarction, unstable angina, and stoke. Exposure to TF upon plaque rupture initiates the extrinsic coagulation pathway. Kapustin et al. found that platelet derived growth factor (PDGF) and tumor necrosis factor-α (TNFα) stimulated release of exosomes from VSMCs (86). This result correlate well with another study which showed that TF is secreted from vesicles (<200 nm) released by VSMCs and this process is regulated by PDGF and TNFα (88). Meanwhile, exosomal PS can bind to coagulation factors. Thus, exosomes released by VSMCs may contribute to vascular thrombosis events (89).

### Roles of Other Cell Exosomes in Atherosclerosis

In 2006, Mayerl et al. analyzed 12 human atherosclerotic specimens collected by von Rokitansky's and reported that T cells were involved in the early pathological process of atherosclerosis (90). Subsequent researchers further confirmed that T cells were present in all stages of atherosclerotic plaque, formatting the basis of adaptive immune response in atherosclerosis (91, 92). Although activated CD4^+^ T lymphocytes infiltrate atherosclerotic plaques, the effects of T-cell exosomes on atherosclerosis-associated cells have not been fully understood. Liudmila et al. isolated exosomes from the supernatant of activated human CD4^+^ T cells and found that endogenous phosphatidylserine receptors mediated the process of T cell exosomes into monocytes, thereby increasing the appearance of lipid droplets containing cholesterol ester and free cholesterol in cytoplasm of recipient cells (93).

Myeloid derived suppressor cells (MDSC) originate from immature myeloid cells under pathological microenvironment. It has been reported that the inflammatory environment and exogenous stimuli that promoted the differentiation of immature myeloid cells can also facilitate the release of exosomes from MDSCs. Deng Z et al. demonstrated that doxorubicin treatment of mammary carcinoma bearing mice led to the induction of miR-126^+^ MDSCs and MDSC miR-126a^+^ exosomes, which could promote tumor angiogenesis (94). Meanwhile, administration of miR-126-5p could rescue EC proliferation and limit atherosclerosis (95), indicating that MDSC-derived exosomal miR-126a might limit the atherosclerotic lesion formation in the same way (96).

Other types of cells in atherosclerotic plaques, such as dendritic cells, can internalize ox-LDL to become foam cells. They increase in atherosclerotic lesions to mediate adaptive cellular immune inflammation reaction in early stage. Mature dendritic cell-derived exosomes mediated activation of NF-κB pathway via abundant TNF-α on their membrane, which increased endothelial cell inflammation and thereby promoted atherosclerosis (97). A recent study demonstrated that adipose-derived mesenchymal stem cells (ADSCs)-derived exosomes could restrain the expression of miR-324-5p which targeted at PPP1R12B in the lesion model for HUVECs. Their work revealed a possible mechanism in which ADSCs-derived exosomes might protect endothelial cells against atherosclerosis (98).

Vascular adventitial fibroblasts play a key role in vascular wall function and structure regulation. It's reported that miR-155-5p transferred by adventitial fibroblasts-derived exosomes could attenuate VSMC proliferation via suppressing angiotension-converting enzyme, which might be anti-atherosclerotic (99).

Exosomes derived from cardiomyocytes in type 2 diabetic rats exerted an anti-angiogenic function by transferring miR-320 into endothelial cells, suggesting that exosomes could promote atherosclerosis by inducing endothelial dysfunction (100). Recently, Xiong Y et al. found a significant upregulation of miR-20b-5p in circulating exosomes in diabetic patients. This miRNA could suppress endothelial cell angiogenesis by regulating Wnt9b/β-catenin signaling (101). However, the cellular sources of these exosomes need further studies.

## Potential Application of Exosomes in the Diagnosis and Treatment of Atherosclerosis

As a natural carrier, exosomes contain abundant biologically active molecules including proteins, mRNAs, miRNAs and lipids, which are cordoned off and protected from degradation by exosomal membrane. Actually, this property of exosomes makes them a novel promising biomarker for diseases. Furthermore, natural or modified exosomes can also serve as therapeutic tools to modulate target cell dysfunction or deliver drug to cells, altering the phenotype and function of the target cells (102).

### As a Biomarker for Disease Detection

Biomarkers have been used for objective measurement and assessment and served as indicators of normal biological function, pathogenic process or pharmacological response to therapeutic interventions (103). Secretion of exosomes increases in response to stress or injury, especially in atherosclerosis patients with vascular injury, inflammation, and prothrombotic state (102). Circulating exosomes have become significant candidates for cardiovascular disease biomarkers. At the same time, the content of exosomes depends largely on the pathological and physiological state of the cells or tissues. The structure of the lipid bilayer keeps the exosome content stable against various enzymes. Therefore, detection of exosomes in plasma may be a novel minimally invasive indicator for early stage of diseases. In addition, extracellular vesicles are similar to their parental cells in many characteristics, such as surface receptors, integrated membrane proteins, cytosolic proteins, mRNAs and miRNAs. Exosomal miRNAs, which may have significantly different circulating levels between healthy subjects and patients with cardiovascular disease, are therefore useful as biomarkers for diagnosis and prognosis.

It's been reported that elevated levels of plasma platelets and/or endothelial extracellular vesicles could be used to predict cardiovascular morbidity and mortality in atherosclerosis patients (104, 105). Kuwabara et al. observed elevated levels of miR-1 and miR-133a in vesicles of patients with myocardial infarction and unstable angina, which could be used as biomarkers for myocardial cell death and injury (106). In addition, serum miR-34a, miR-192, and miR-194 are elevated in patients with acute myocardial infarction, especially in CD63-positive exosomes, which is associated with a high risk of ischemic heart failure 1 year after myocardial infarction (107). Recently, a study reported a higher expression of miR-30e and miR-92a in exosomes from serum in patients with coronary atherosclerosis compared with healthy subjects. MiR-30e and miR-92a targeted ATP binding cassette (ABC) A1 and downregulated its expression, subsequently regulating cholesterol metabolism. Therefore, the levels of miR-30e and miR-92a in exosomes from serum could be a novel diagnostic biomarker for atherosclerosis (108). In addition, it's reported that specific kind of circulating exosomal miRNAs such as miR-122-5p, miR-27b-3p, and miR-24-3p etc., could be a novel predictor for recurrent ischemic events in intracranial atherosclerosis (109).

### Potential Application of Exosomes to the Treatment of Atherosclerosis

As a natural vesicle isolated from tissues or cells, exosomes have biocompatibility, bio-barrier permeability, low toxicity and low immunogenicity, which shows that exosomes have potential therapeutic effects in diseases (24, 110).

Exosomes, delivering miRNAs for repair of damaged tissues caused by atherosclerosis (such as delivery of atheroprotective miRNAs) or anti-miRs (e.g., antisense miRNAs) for clearance of pro-atherosclerotic miRNAs are considered as a promising carrier tool to be used for the treatment of atherosclerosis. The potential miRNAs are mainly miR-126 (endothelium-specific and atheroprotective) and miR-145 (VSMC-specific and atheroprotective) (111).

Exogenous miRNAs, siRNAs, and even drugs can be encapsulated in natural exosomes or engineered exosomes. Mature techniques for encapsulation of therapeutic miRNAs or siRNAs in exosomes can be used through the following methods: (1) donor cells can be co-transfected with two types of plasmids or viruses (112); (2) miRNAs or siRNAs can be directly loaded to purified exosomes through electroporation (113, 114); (3) transient transfection of miRNAs using commercial transfection reagents (115); (4) other loading methods, such as permeabilization by saponin, sonication or extrusion can improve loading efficiency, but still need further verification (116). For example, Wu G et al. discovered that M2 macrophage-derived exosomes electroporated with FDA-approved five-administration hydrochloride exhibited excellent inflammation-tropism and anti-inflammation effects and could be used for AS imaging and therapy (117). Techniques for assembling drugs into exosomes are also evolving. There are currently three different methods for drug loading into exosomes: (1) direct incorporation of small molecule drugs into purified exosomes such as lipophilic small molecules, low molecular weight antioxidants and anticancer agents (113, 118, 119); (2) loading the drug into the donor cell which secretes exosomes thereby loading the drug into the exosomes (116, 120); (3) direct transfection of the drug-encoding DNA into donor cells resulting in drug expression and sorting into exosomes (120). Kalani et al. loaded curcumin into donor mouse brain endothelial cells by direct infiltration. Curcumin-loaded exosomes isolated from endothelial cells penetrated the blood-brain barrier well and could reduce oxidative stress and MMP-9 levels, as well as improve endothelial cell permeability to ultimately reduce dysfunction of endothelial cell induced by hyperhomocysteinemia (121). In general, the characteristics of technology, function and safety for exosomes have not been fully understood. The therapeutic significance of exosomes in atherosclerosis remains to be further studied.

Recently, a study provided a new strategy for the treatment of patients with familial hypercholesterolemia (FH) and managing atherosclerosis. They used low-density lipoprotein receptor (Ldlr)-deficient mice (Ldlr^−/−^ mice) as a model of FH and found that exosomes-mediated Ldlr mRNA delivery could robustly restored Ldlr expression in the Ldlr^−/−^ mouse model. Significantly, serum LDL-cholesterol levels were lowered and the number and size of atherosclerotic plaques were reduced (122). In addition, *in vivo* experiments showed that mesenchymal stem cell-derived exosomes with miR-145 could downregulate JAM-A expression and reduce atherosclerosis plaque size, suggesting the role of exosomes derived from MSCs containing miR-145 in the treatment of atherosclerosis (123). Chrysin could attenuate the expression of miR-92a in exosomes derived from human coronary artery endothelial cell and counteract the inhibitory effect of miR-92a on the expression of KLF2, and play an atheroprotective role (124).

## Conclusions

As a new and effective intercellular communication tool, exosomes have attracted more and more attention in the field of biology, including atherosclerotic cardiovascular diseases. Exosomes envelop various biomolecules according to pathological/physiological conditions and different cellular sources. In most cases, exosomes mainly promote the progression of atherosclerosis. However, there are also studies showing the atheroprotective role of exosomes, indicating the multifuctional property of exosomes in atherosclerosis. To date, there are still many problems to be solved regarding exosomal biology, such as exosome formation, release, internalization and clearance. Besides, the molecular mechanisms of exosome-based intercellular communication associated with atherosclerosis are expected to be elucidated.

As for the clinical application, the assessment of circulating exosomes as a diagnostic and prognostic biomarker for cardiovascular risk has just started. Several technical limitations still exist, such as the lack of gold-standard method for exosome isolation as mentioned earlier and the influence of confounding factors like disease specificity the presence of comorbidities. It needs more research to validate the relationship between disease progression and exosome-associated biomarkers (such as miRNAs carried in exosomes) and figure out the value of exosomes for diagnosis and prognosis. In addition, the precise cellular origin, package and secretion mechanisms of biomarkers carried by exosomes and their functional roles remain to be studied.

Because exosomes are natural carriers of biologically active molecules, they can be an attractive therapeutic tool. They may act as carriers for drugs or siRNAs to control gene expression and accelerate disease recovery, or to carry excess lipids at the plaque to expel atherosclerotic plaques, thereby slowing the onset and progression of the disease. In addition, although some meaningful findings have been made on the prognosis and treatment of vascular diseases, research on exosome-based treatment methods is still limited, so further basic medical research is needed before applications.

## Author Contributions

JY wrote the initial version of the manuscript. BL made supplements to the manuscript based on the latest studies. JF revised the manuscript. All authors contributed to the article and approved the submitted version.

## Conflict of Interest

The authors declare that the research was conducted in the absence of any commercial or financial relationships that could be construed as a potential conflict of interest.

## Publisher's Note

All claims expressed in this article are solely those of the authors and do not necessarily represent those of their affiliated organizations, or those of the publisher, the editors and the reviewers. Any product that may be evaluated in this article, or claim that may be made by its manufacturer, is not guaranteed or endorsed by the publisher.
